# Recent Advances in Drying Technologies for Orange Products

**DOI:** 10.3390/foods14173051

**Published:** 2025-08-29

**Authors:** Xindi Tan, Wenzhan Jiang, Jiaying Su, Fanqianhui Yu

**Affiliations:** 1 Haide College, Ocean University of China, Qingdao 266100, China; xindi_tan@stu.ouc.edu.cn (X.T.); wenzhan_jiang@stu.ouc.edu.cn (W.J.); sujiaying@stu.ouc.edu.cn (J.S.); 2 Department of Computer Science and Technology, Ocean University of China, Qingdao 266100, China

**Keywords:** orange products, drying technologies, bioactive compounds, high-value utilization

## Abstract

Oranges are popular worldwide, due not only to their refreshing taste but also to their high content of bioactive compounds. The main phytochemicals in oranges are phenolic compounds, vitamins, and carotenoids, which contribute to their antioxidant and anti-cancer activities. Various drying methods are used to remove the high moisture content in orange products to extend their shelf life. This review summarizes and compares the effects of different drying methods such as hot air drying, freeze drying, vacuum drying, spray drying, microwave drying, solar drying and innovative pretreatment on the physicochemical quality of orange products including slices, peels, and by-products. It lists the key parameters, advantages, and disadvantages of drying methods, as well as a decision tree for “product type-constraints-recommended drying method with pretreatment”. For example, the results indicate that vacuum microwave drying is effective in drying orange slices, and control techniques are employed to assist the drying process. Freeze drying preserves more phytochemicals in orange peels and their by-products, which results in higher antioxidant activity. Pretreatments like pulsed electric field and ozone enhance drying efficiency and phytochemical retention. Different drying methods are adopted to treat different by-products. This work can be used as a guide for selecting the optimal drying technique to balance efficiency, nutritional quality, and industrial scalability for different orange products.

## 1. Introduction

Oranges are one of the most popular and high-yielding fruits in the world. From 2024 to 2025, global orange production reached approximately 45.22 million metric tons (Mt). Brazil was the leading producer with 13 Mt (29% of global production), followed by China with 7.62 Mt (17%), Mexico with 4.97 Mt (11%), and the European Union with 1.36 Mt (3%) [1]. Oranges are valued not only for their vibrant flavor, but also for their impressive nutritional benefits, including a high content of vitamin C, polyphenols like hesperidin and naringin, and flavonoids such as quercetin and rutin [2,3,4]. These components collectively contribute to antioxidant, anti-inflammatory, and immunomodulatory properties, positioning oranges as potentially playing a role in mitigating chronic diseases such as cardiovascular disorders [5,6] and diabetes [7,8].

Despite their nutritional richness, fresh oranges face significant postharvest challenges due to their high moisture content (approximately 85–88%), which accelerates microbial spoilage and enzymatic degradation [9]. The perishable nature of fresh produce results in significant economic losses, estimated at 20–50% of total yield in developing countries, during storage and transportation [10]. To address these limitations, drying technologies have emerged as a critical solution to extend shelf life while preserving bioactive compounds [11]. Alternatively, dried and powdered oranges may be used on their own or as an additive in many different food products [12]. Beyond shelf-life extension, drying also facilitates the utilization of orange by-products. For instance, Oyebola et al. [13] found that peels account for 45–50% of the sweet orange (*Citrus sinensis*)’s weight and contain concentrated levels of nutrients and non-nutritive phytochemicals such as minerals, pectin, and antioxidants [14,15].

Currently, dried orange products on the market include snackable fruit chips, tea blends, and natural flavoring agents, which are produced by different drying techniques. For example, hot air drying is the most commonly used traditional method for drying orange slices. This technique has the advantages of low cost and user-friendliness, but it often causes nutrient degradation and uneven moisture removal due to long processing times [7]. In contrast, techniques such as freeze-drying, microwave drying, and vacuum drying have gained recognition for retaining thermolabile nutrients, improving energy efficiency, and enhancing sensory attributes [16,17]. Moreover, microwave drying, pulsed electric field pretreatment, or radio frequency in combination with hot air drying can synergistically improve efficiency, further promising to optimize the balance between operational costs and product functionality [18,19,20]. Other innovative technologies, such as electrohydrodynamics, solar drying, and infrared drying, offer distinct advantages. Specifically, electrohydrodynamics can reduce drying temperature and time, solar drying uses renewable heat, and infrared drying raises heat transfer coefficients [21,22].

Previous reviews have summarized various aspects of citrus drying, but there remains a significant gap in guiding method selection for specific orange products. For instance, the review of Liu et al. (2022) focused on the phytochemical and nutritional characteristics of *Citrus* L. fruits, but lacked detailed comparisons of drying technologies’ impacts on these qualities [23]. Li et al. (2023) reviewed utilization methods of orange pomace and peel but did not include other form of orange products [24]. Additionally, the review of Abd Elghani discussed only spray drying for juice powders [25]. Based on the above, this review summarizes the effects of different drying technologies on the physicochemical properties of orange products (including slices, peels, and other by-products), providing valuable guidance for selecting appropriate drying methods for various orange-derived products under different production requirements. This work aims to inform industry practices and inspire further research on how to maximize the functional value of orange-derived products as competitive ingredients in nutraceutical and food applications.

## 2. Review Methods

(1) Databases and timeframe: Scopus/Web of Science/PubMed, 2019–2025.
(2) Search strings: combine orange/citrus/*Citrus sinensis*/orange products/orange slices/orange peel/orange waste/citrus tea with hot air drying/freeze drying/vacuum drying/microwave drying/microwave vacuum drying/infrared drying/pulsed electric field pretreatment/ozone pretreatment/spray drying/ultrasound drying.
(3) Inclusion/exclusion: peer reviewed; English/Chinese if included; exclude conference abstracts without data; include only studies that report method parameters and at least one of (ΔE, TPC, AAC, Antioxidant activity, total polyphenol content, Deff, EO yield). Include only studies that report dried oranges in food applications.

## 3. Properties of Oranges

Oranges are made up of water (~86%), with significant amounts of sugar (~8.6%), dietary fiber (~2%), protein (~0.91%), and minimal fat (~0.15%). Orange peels have a slightly lower water content (70–78%) [26,27,28,29]. Oranges are rich in essential micronutrients, such as vitamin C (59.1 mg/100 g), potassium (166 mg/100 g), and calcium (43 mg/100 g) [30]. Remarkably, phytochemicals are abundant in oranges, which generally have a higher concentration in orange peels than the flesh and seeds [31,32]. These phytochemicals consist of phenolic acids, flavonoids, carotenoids, terpenoids, and tannins, which exhibit strong antioxidant and anti-inflammatory properties [31,33]. Additionally, the oil glands in orange peel, containing various volatile compounds, impart the characteristic aroma of oranges [34]. These bioactivities collectively contribute to the growing interest in the utilization of whole orange components, especially the peels, in food, pharmaceutical, and cosmetic industries [35,36].

### 3.1. Phenolic Compounds

Phenolics in orange peels consist of numerous chemicals including phenolic acids and flavonoids, which are secondary metabolites synthesized by plants as a defense mechanism against ultraviolet radiation and pathogens [37]. These compounds contribute significantly to the antioxidant and anti-inflammatory properties of oranges [31]. Phenolic acids are composed of derivatives of benzoic acids (gallic acid, vanillic acid, and 4-hydroxybenzoic acid) and cinnamic acids (ferulic acid, caffeic acid, and sinapic acid) [36,37,38]. The total phenolic contents (TPC) of fresh orange peels vary from different extraction methods. Studies have shown that methanol is an effective solvent for extracting phenolic acids [31], with concentrations ranging from 23.48 to 35.6 mg gallic acid equivalent (GAE)/g dry matter (dm) [28,32,39,40]. However, Liew et al. (2018) determined that 70% acetone/water extract showed a higher TPC (38.24 mg GAE/g) than methanol [37]. Using ultrasound-assisted extraction can achieve 8.7–34.71 mg GAE/g [41,42,43]. In addition, growth stages of oranges also affect TPC, as Hou et al. (2021) found that TPC exhibited a decreasing trend from 18.83 mg to 5.69 mg GAE/g during ripening [44].

Flavonoids are the predominant subclass of phenolics in oranges, and are subdivided into flavanones, flavones, flavonols, polymethoxylated flavones, isoflavones, and anthocyanins, contributing significantly to antioxidant activity [39,45,46]. Polymethoxylated flavones (PMFs) are a unique class present in *Citrus* species, especially in orange peels [36], and have been found to have anti-cancer properties [47]. Another species-specific flavanone, hesperidin, showed the highest concentration in flavonoids [48], which can contribute to multiple bioactivities. As to the content, total flavonoids content (TFC) in orange fruits is often presented using catechin equivalent (CE). Elkhatim et al. [32] found that orange peels contained the highest amounts of flavonoids (83.3 mg CE/g, using ethanol as the extraction solvent) compared to peels of other citrus fruits, which was significantly higher than that in the inner part (pulp and seeds). In contrast, Liew et al. [37] found TFC ranging from 1.9 to 5.51 mg CE/g with 50% (*v*/*v*) acetone/water solvent extraction yielding the highest TFC, while Abdelazem et al. [31] extracted 0.79 to 16.68 mg CE/g with water extraction yielding the highest TFC. This variation may be due to different orange species and growth stages. Chen et al. investigated the flavonoid content of five cultivars of *Citrus sinensi* and found that TFC ranged from 2.81 mg to 5.52 mg CE/g [49]. Similar to phenolic compounds, flavonoids in navel orange pulp also reduced with ripening from 2.56 mg CE/g at the young fruit period to 0.33 mg CE/g.

### 3.2. Carotenoids

Carotenoids are fat-soluble isoprenoids pigments responsible for the orange color of orange peels, which help neutralize free radicals within cell membranes, protecting cells from oxidative damage [50]. They can be classified into three categories: carotenes (purely hydrocarbons), xanthophylls (oxygenated derivatives), and apocarotenoids [46]. Among them, β-carotene is a major carotene with a linear, symmetrical structure and multiple double bonds. As a precursor to vitamin A, β-carotene plays a crucial role in human health by supporting eye function and acting as a potent antioxidant [33]. In addition to β-carotene, oranges contain various xanthophylls such as violaxanthin isomers (notably 9-cis-violaxanthin), lutein, zeaxanthin, and β-cryptoxanthin. These pigments vary in concentration across different parts of the orange [46]. Similar to the phenolic compounds, orange peels contain more carotenoids than the inner part. Elik et al. (2023) extracted 599.50 ± 3.28 µg/g dry weight from orange peels [18]. Lux et al. (2019) found that the flavedo of oranges contained 126.05 µg/g compared to 13.54 µg/g in the endocarp [51] and Chen et al. (2023) had similar results [52]. However, the carotenoid content is higher in mature fruits [51].

### 3.3. Vitamins

Vitamin C is abundant in oranges and plays a critical role in human health through its antioxidant capacity [50]. It is a potent antioxidant that neutralizes free radicals and reduces oxidative stress, thereby protecting cells from damage, and has potential anticancer effects. Vitamin C content varies in different parts of the fruit [53]. The flavedo contains the highest total vitamin C concentration among the flavedo, albedo, and juice, with 85.5 mg/100 g, 16.3 mg/100 g, and 33.1 mg/100 g, respectively. The vitamin C in juice exists entirely as L-ascorbic acid (reduced form), whereas flavedo and albedo contain 70.8% and 51.0% dehydroascorbic acid (oxidized form), respectively [53]. This is in agreement with the findings of Alos et al. (2021), where the total ascorbic acid content of the flavedo ranged from 73 to 407 mg/100 g and was higher than that in the pulp [54]. Compared to other citrus fruits, orange has the highest vitamin C content determined by indophenol titration (58.30 mg/100 g) and high-performance liquid chromatography (HPLC) (43.61 mg/100 g) [54]. However, Elkhatim et al. found that the vitamin C content of 110.4 mg/100 g in orange peel and 89.8 mg/100 g inside the peel was slightly lower than grapefruit [32].

Vitamin E is a lipid-soluble antioxidant in oranges and its content in sweet oranges is 0.18 mg/100 g [34,55]. The antioxidant mechanism involves interrupting lipid peroxidation chain reactions by donating hydrogen atoms from its chromanol ring to lipid peroxyl radicals, generating stable tocopheryl radicals that are recycled back to active vitamin E via redox systems like vitamin C or glutathione [33]. This enhances the activity of antioxidant enzymes, reduces lipid peroxidation, and supports the maintenance of hematological stability under conditions of oxidative stress [56]. Oranges also contain vitamin A, which is produced from β-carotene and is essential for maintaining overall health.

### 3.4. Health Benefits and Side Effects

Oranges are a main source of nutrients and important non-nutrient phytochemicals with numerous health benefits, including boosting immunity, cardiovascular protection, and reducing the risk of chronic diseases such as diabetes and obesity [57]. Orange peel in particular is rich in flavonoids, phenols, and antioxidants that help control inflammation, which can further reduce obesity-induced dysfunction [58]. Traditional Chinese medicine has long utilized orange peel for treating digestive disorders, respiratory conditions, and metabolic issues like obesity and hypertension [59,60]. In addition, researchers have found that the consumption of orange juice may protect healthy individuals from hyperuricemia and gout [61], and lower cholesterol and LDL levels [62]. Meanwhile, there is little evidence showing significant side effects of orange consumption for healthy people. However, high consumption of orange juice may lead to an increased risk of gout due to its high fructose content [63]. In addition, the high acidity of citrus fruits can also trigger gastroesophageal reflux symptoms [64]. The USDA identifies oranges and 100% orange juice as nutrient-dense foods and recommends ~2 cup-equivalents of fruit/day [65].

## 4. Current Drying Technologies

### 4.1. Hot Air Drying and Freeze Drying

Hot air drying (HAD) is a common food drying technique with low investment and operating costs [66]. It is based on the principle of using hot air as a heat and mass transfer medium to transfer heat to the food, causing internal moisture to evaporate and be carried away by the airflow [67], thus controlling the temperature and relative humidity of the drying medium is crucial. Typically, HAD at 60–80 °C negatively affects the sensory attributes such as color, texture, aroma, and the nutritional properties of some foods [2,68,69], but researchers have recently found that hot-air-dried orange dark tea contains higher levels of polyphenols, flavonoids, hesperidin and limonin, and less astringent bitterness [70].

Freeze drying (FD) is also a popular drying method that removes water from food through a process of freezing and sublimation. The food is frozen at low temperatures to turn the moisture inside into solid ice. The environment is under vacuum and at low pressure below the triple point pressure of water, the solid ice can be converted directly into water vapor without first becoming liquid [71]. Compared to HAD, a significant advantage of FD is its ability to preserve the food’s inherent qualities such as color, aroma, flavor, and nutritional value [72]. This method is particularly beneficial for heat-sensitive products, as it avoids the changes that may occur during the heating process in traditional drying methods. In addition, freeze-dried foods can be easily reconstituted with water to quickly restore their original texture and flavor [72].

However, the FD process is usually time-consuming and requires specialized equipment, making it more costly compared to other dehydration methods. For instance, drying blueberries may require 22 h [73] to reach the desired moisture content, i.e., from an initial 6.892 g/g dw to 0.12 g/g dw. Meanwhile, mango peels can take as long as 72 h [74] to reach the ideal dry state, with moisture content reducing from 3.532 g/g dw to 6.57% g/g dw. Additionally, while pretreatment methods such as CO_2_-laser micro-perforation technology can improve drying efficiency, the reduction in drying time is often marginal, e.g., drying time is reduced from 26.7 h to 22.3 h [75]. These examples highlight the challenges of FD in terms of time and cost; despite its advantages in preserving product quality, additional improvements need to be explored in the future.

### 4.2. Vacuum Drying and Pulsed Drying

Vacuum drying (VD) is a drying technique conducted in a low-pressure environment. The basic principle involves reducing the pressure by creating a vacuum to lower the boiling point of water, enabling evaporation at lower temperatures [76]. Due to the low concentration of oxygen in the vacuum environment, oxidation reactions or browning are substantially suppressed, thus maximizing the retention of the active ingredients and quality of the product [77].

However, exposing products to low pressure for long periods of time may cause irreversible and severe deformation [78]. To avoid this problem, more and more scientists are focusing on pulsed vacuum drying (PVD). PVD works by periodically altering the vacuum level during the drying process to make the pressure changes more uniform to minimize stress concentrations [78]. Additionally, periodic pressure variations create a “tunneling effect”, which enlarges or interconnects the microporous structure within the food matrix [79]. This optimization of the microporous structure facilitates more uniform moisture evaporation, reducing localized over-drying or under-drying and further mitigating deformation risks. Moreover, moisture is efficiently removed through capillary channels generated by pressure pulsation, avoiding localized expansion or contraction due to moisture retention and significantly reducing the likelihood of deformation [80]. Together, these mechanisms make PVD particularly effective for maintaining the structural integrity of heat-sensitive materials during drying. In recent years, PVD has been employed in the drying of lemon slices [81], orange slices [76,78], sea buckthorn berries [82], and winter jujube [83], etc. For example, Xu et al.’s team [76] used PVD with operational windows of vacuum pressure levels (20.0, 15.0, and 10.0 kPa) and pulsed vacuum ratios (5:10, 5:15, and 5:20 min vacuum time to atmospheric pressure time) to dry bitter orange (*Citrus aurantium* L.) slices. They found that overly aggressive pressure swings (e.g., 10.0 kPa with a 5:20 min ratio) led to higher color degradation (ΔE = 10.39) and browning index (BI = 32.73 for peel), suggesting potential structural stress. The optimal balance was achieved at 10.0 kPa with a 5:15 min ratio, minimizing quality loss while maximizing drying efficiency. Excessive pressure fluctuations beyond this range could risk puffing or collapse, as implied by the increased ΔE and BI in extreme conditions.

### 4.3. Spray Drying

In order to extend shelf life, expand the market for functional foods, and reduce waste of agricultural and sideline products, many juices with added nutrients (e.g., prebiotics) have been spray dried into powder in recent years [84,85]. The mechanism of spray drying involves the atomization of a liquid feed into fine droplets, rapid evaporation of moisture through contact with hot air (inlet temperature is generally set at 140–200 °C and outlet temperature varies from 40 to 90 °C) [85], and the subsequent formation of dry particles, which are then separated and collected, resulting in a powdered product with controlled moisture content and particle size. However, high content of low molecular weight sugars (e.g., fructose, glucose, sucrose) and organic acids in fruit juices contribute to sticky and caking powders during drying due to their low glass transition temperature (Tg), necessitating the use of high molecular weight additives like maltodextrin, Arabic gum, crystallized starch, or cellulose as carriers to raise Tg in the feed solution and further improve the powder yield [86,87]. Specifically, Tg refers to the temperature at which an amorphous system changes from a glassy to a rubbery state. Theoretically, in the glassy state, the high viscosity of the matrix prevents diffusion-controlled reactions from occurring. The Gordon–Taylor equation is an empirical formula that predicts the Tg of mixtures based on the Tg of each component in the mixture and their mass fractions. For example, the results from Yousefi et al.’s study [87] shows that spray drying pomegranate juice without carriers leads to material adhesion on the chamber walls and cyclone separator, and a hard glassy film forms on the walls as the spraying time increases. The Tg of spray-dried pomegranate juice produced using maltodextrin (12%) without cellulose and with 3% cellulose increased from 40.0 ± 3.30 °C to 59.9 ± 1.20 °C. These results indicate the importance of adding carriers.

### 4.4. Microwave Drying and Infrared Drying

Generally, microwave and infrared drying technologies have been increasingly combined with other drying methods to improve the efficiency and quality of fruit drying processes. Microwave drying (MWD) has been paired with techniques such as HAD and VD to improve drying rates and preserve nutritional properties. For instance, microwave-assisted convective drying has been applied to pear slices, which significantly reduced drying time but resulted in color loss of the slices [88]. Similarly, microwave–vacuum drying at a lower microwave power level and higher vacuum level (low absolute pressure) has been utilized for sohiong (Prunus nepalensis) fruit, resulting in improved retention of color, anthocyanin, DPPH scavenging activity, and phenolic components [89]. In addition, due to the low oxygen content provided by the vacuum, the oxidation of molecules was prevented during drying, resulting in dried products with higher nutritional value [89].

Infrared drying (ID) is combined with other drying techniques to optimize fruit drying most of the time. For example, in infrared-assisted FD, infrared radiation provides energy for sublimation instead of electric heating plates, which boosts heat transfer rates, cuts energy consumption, shortens drying time, and lessens quality degradation [90]. For the infrared-assisted solar dryer, the integration of IR heating addresses the limitations of inconsistent drying temperatures and prolonged drying times in traditional solar drying, resulting in higher drying rates (0.95 kg.water/kg.dry solid.h), improved moisture diffusivity (2.59 × 10^−10^ m^2^/s), and better retention of quality attributes like color, phenolic content, and antioxidant activity in pineapple slices [91]. Research has shown that infrared-assisted FD improves the crispness and structural integrity of banana slices [92], reduces drying time and energy consumption for strawberry snacks [93], and optimizes the operational efficiency of sweet potato slices by extending pre-drying times and temperatures, although color and hardness may decrease as drying temperatures increase from 40 °C to 60 °C in mid-ID [94]. Moreover, Near-infrared (NIR) radiation is often used for drying berries such as açai puree, blackberries, and strawberries, probably because berries are typically dried whole, rather than sliced [95]. NIR allows for the rapid and thorough drying of whole berries, which maintains their aesthetic appearance, high nutritional content, and strong rehydration capacity [95,96,97].

Furthermore, infrared-assisted HAD, infrared-assisted MWD, and infrared-assisted VD have been developed to address the drawbacks of traditional drying methods. For example, infrared-assisted HAD can improve product quality and reduce energy consumption, but the optimal combination needs to be determined [98]. Infrared-assisted MWD can solve the uneven drying problem of MWD, such as black tea drying [99]. And infrared-assisted PVD not only improves drying efficiency but also enhances product quality (e.g., drying blueberries), which has been proven to have great potential [100].

### 4.5. Solar Drying

Solar drying (SD) is gaining popularity due to its being environmentally friendly by using renewable resources, easy availability in sunny locations and low maintenance cost [101,102]. In the drying process, solar energy is harnessed through a solar collector [103]. The collector absorbs solar radiation and converts the light energy into heat, which is then used to heat the air. The heated air is directed into the drying chamber by natural convection or forced convection with the aid of a fan [103,104]. The heat from the air causes the moisture within the material to be heated and evaporated, just like other traditional drying technologies. To maintain a low-humidity environment in the drying chamber and facilitate further drying, the evaporated moisture is expelled through the chamber’s exhaust system. This continuous process of heating, evaporation, and moisture removal enables the efficient drying of the material.

Teguia’s team demonstrated that the mass flow rate significantly impacts the solar drying efficiency of orange slices, with the highest tested flow rate of 0.033 kg/s achieving the fastest drying performance [103]. This optimal flow rate enhanced heat transfer in the solar collector, resulting in higher outlet air temperatures that maintained elevated and stable conditions in the drying chamber, thereby accelerating moisture evaporation [103]. Moreover, SD doubles the energy efficiency to 20% to 30% [103]. SD has been successfully applied to strawberry tree fruits [105], date fruits [106], mangoes [107,108], pineapples [108], potatoes [109,110,111], apples [111,112,113], and eggplants [111].

### 4.6. Ozone Pretreatment

Ozone (O_3_) pretreatment has emerged as a promising technique in food drying, offering significant advantages in terms of processing efficiency and product quality. Ozone exerts its effects primarily through oxidation, which can disrupt microbial cell walls and membranes, thus reducing microbial load, which makes it particularly effective in decontaminating food products before drying [114]. It has been demonstrated that ozone has an effect on total bacterial, Enterobacteriaceae, coliform, yeast, and mold populations [115]. In addition, ozone can degrade pesticide residues through oxidative reactions and improve the safety of dried foods [114]. For instance, Bae et al. [116] reported a 57.9% reduction in pesticide residues (e.g., dimethomorph and thiamethoxam) in dried red peppers following ozone treatment [116]. As a strong oxidizing agent, ozone can alter the microstructure of food surfaces through oxidation, enhance moisture diffusion, and accelerate the drying process [117]. However, the effectiveness of ozone depends on factors including concentration, exposure time, and the specific food matrix [114,117]. Furthermore, unlike conventional treatments such as chlorine, ozone pretreatment improves food safety without leaving harmful residues [118].

Specifically, a notable example of ozone pretreatment is its application to orange peel. Bechlin et al. [27] found that exposing orange peel to ozone (4–40 μg/L for 2 h) before convective drying at 40–60 °C significantly reduced phenolic content (6.89–8.05 mg GAE/g) compared to untreated controls (9.81 mg GAE/g). The high reactivity of ozone can lead to the degradation of sensitive nutrients, such as proteins and phenolic compounds, and may alter the flavor and aroma of dried products [114]. This highlights the need for optimized conditions to balance drying efficiency and bioactive compound retention. Moreover, other studies have demonstrated that ozone pretreatment also improves drying efficiency and color retention of apples [119], as well as the antioxidant activity of lemon peel, although some oxidative browning occurs [120]. These results illustrate ozone’s dual role in enhancing drying processes while potentially affecting food quality. In addition, ozone is unstable at room temperature and must be generated on-site, and prolonged exposure to ozone poses health risks including respiratory problems, and therefore requires careful handling and regulation [117].

Based on this, we summarized the application characteristics of using the above drying techniques in dried orange products, as shown in Table 1.

## 5. Dried Orange Products

**Figure 1:**
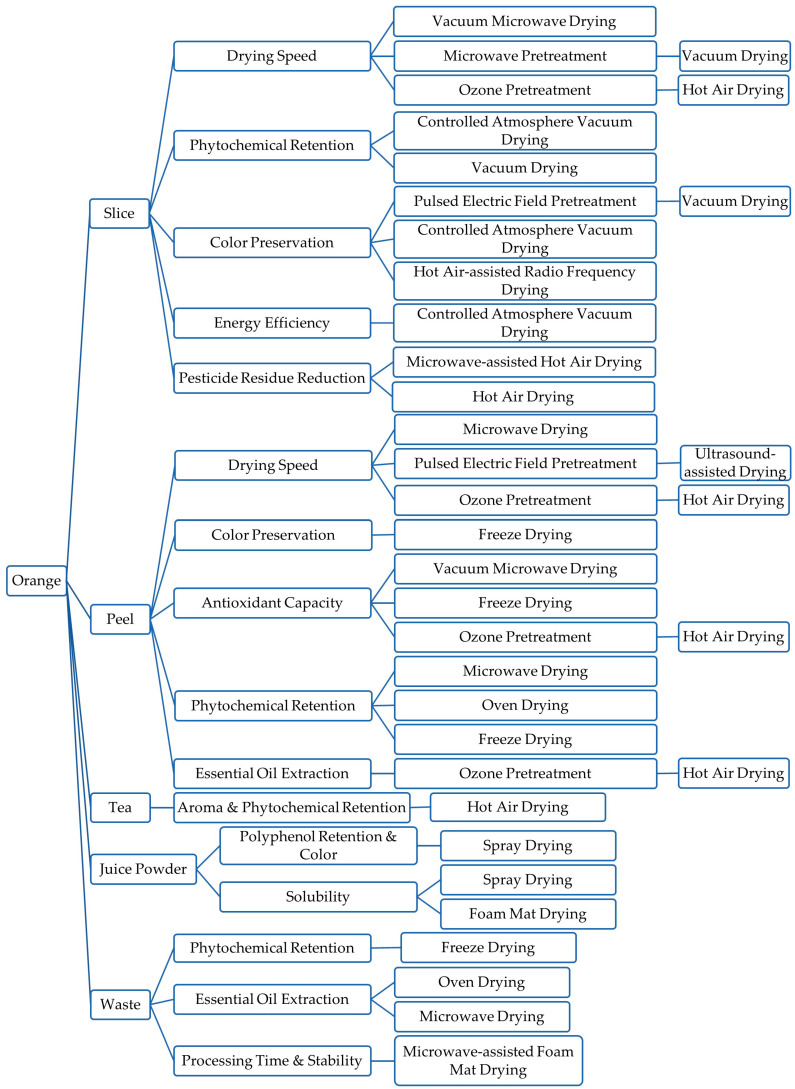
Decision tree of “product type-constraints-recommended drying method with pretreatment”.

According to the literature review over the past five years, we summarized orange-derived products prepared using different drying technologies and drew a visual decision tree of “product type-constraints-recommended drying method with pretreatment”, as shown in Figure 1.

### 5.1. Orange Slices

Dried orange slices are dehydrated fruit flesh with minimal peel. Compared to fresh oranges, dried orange slices have a sweeter and more acidic taste and a crispier texture. They are the most common and popular dried orange product on the market, and can be produced by different drying techniques. For example, Bozkir’s [2] study compared the effects of three drying techniques, HAD, vacuum infrared drying (VID), and vacuum microwave drying (VMD), on drying efficiency and product quality of orange slices. The results showed that VMD (operating power: 250–1000 W, vacuum pressure: 0–1000 mbar) significantly reduced the drying time of orange slices to 69 min at 70 °C, compared to 180 min for HAD and 92 min for VID, representing a reduction of approximately 62% and 25%, respectively, relative to the conventional methods. TPC and ascorbic acid content (AAC) of VMD-treated samples were significantly higher than those of HAD- or VID-treated samples. In terms of visual quality, the minimal ΔE was also better than that of VID and HAD-treated samples.

Researchers also compared VD against microwave-pretreated vacuum drying (MWVD) for preserving Valencia oranges. Compared to VD, MWVD was faster, reducing drying times by 23.81%, especially at higher temperature (80 °C) and lower pressure (15 kPa). While MWVD was effective in preserving TPC, retaining up to 1040.95 mg GAE/100 g dw, it was less gentle on the heat-sensitive AAC (initial value of 45.18 ± 0.10 mg/100 g dw), which suffered a 59.34% loss (reduced to ~18.38 mg/100 g dw). VD, although slower, was gentler on AAC. In terms of antioxidant activity, VD scored higher in FRAP and CUPRAC assays, but both methods caused significant drops in DPPH activity (up to 35.18% loss), and it was observed that VD caused less decrease in total antioxidant capacity (TAC). Color-wise, MWVD kept the slices relatively vibrant, with the least ΔE (5.80) and minimal darkening, while VD at 70 °C−30 kPa made the slices noticeably duller (ΔE 11.62) [69]. Based on the above two studies, we can see that VMD/MWVD has good performance in many aspects.

To further improve the drying effect, researchers began to attempt to introduce other control techniques in the vacuum environment, such as pulsed vacuum and gas-assisted. In the study by Xu et al. (2021), PVD was compared with VD and atmospheric pressure drying (APD) for drying bitter orange slices [76]. The experiments were conducted at 75 °C with different pressures and pulsed vacuum ratios. The efficiency of PVD was remarkable compared to APD, with a 72.9% reduction in final moisture content (MC), which was lowest at 0.076 g/g dry basis at 10.0 kPa, 75 °C, and a 5:15 min ratio. The effective diffusivity (Deff) for PVD ranged from 1.329 to 2.991 × 10^−10^ m^2^/s, which was superior to that of VD. In terms of color, PVD minimized degradation with an average ΔE of 5.15 and browning index (BI) of 14.37, significantly better than VD and APD. Moreover, Homayounfar et al. (2023) investigated the drying of orange slices under controlled atmosphere (CAtm, nitrogen injection) versus normal atmosphere (NAtm) in a vacuum setting [125]. Drying was carried out at 45–85 °C and 40–80 kPa. CAtm significantly improved drying efficiency, with a 225% increase in effective moisture diffusivity and a 54% reduction in energy consumption compared to NAtm. It also provided better retention of antioxidant properties, with CAtm showing DPPH activity 1.92 times higher than control under optimal conditions and maintaining 26% higher AAC by minimizing oxygen exposure. Compared to NAtm, CAtm shows less color change and a 260% reduction in Δa*. Both studies demonstrated that integrating pulsed vacuum or controlled gas atmospheres into VD improves drying kinetics, moisture removal, and quality retention of orange slices. PVD optimizes drying efficiency and color preservation, while CAtm improves antioxidant retention and reduces energy consumption, which highlights the benefits of combining vacuum environments with dynamic process control.

Additionally, recent advancements in HAD technology have revitalized the field. The combination of auxiliary heating methods has proven to improve energy efficiency and drying uniformity. In a study by Ismail and colleagues (2024), the researchers explored hot air-assisted radio frequency drying (HA-RFD) for orange slices [126]. They compared this method with conventional HAD and FD. HA-RFD was conducted at 47 °C with a 70 mm electrode gap using three orange slices, each 12 mm thick. The results were impressive, as HA-RFD reduced drying time by 67%, from 1170 min for HAD to just 390 min. HA-RFD also achieved a higher Deff of 2.48 × 10^−9^ m^2^/s, almost double the 1.11 × 10^−9^ m^2^/s of HAD. In terms of nutrient preservation, HA-RFD retained 90.82% of TPC (861 ± 10 mg per 100 g dry weight), and 60.28% of AAC (173 ± 3 mg per 100 g dry weight), which was superior to that of HAD. Meanwhile, the antioxidant activity of HA-RFD samples was 59.65 ± 1.50% as measured by DPPH, which was higher than that of HAD samples (44.00 ± 0.89%), but lower than 67.58 ± 1.88% for the FD sample. Color analysis revealed that HA-RFD samples had the smallest color difference (ΔE = 4.49 ± 0.10) compared to fresh slices, whereas FD samples had a larger ΔE (16.32 ± 0.10), suggesting that the color change was more significant. The results also showed that increasing the sample thickness increased the drying rate of HA-RFD, which can be attributed to enhanced radio frequency (RF) energy coupling. As sample thickness increases, the reduced air gap between the sample surface and top electrode intensifies the electric field [127], leading to more efficient volumetric heating. This effect overcomes the conventional thickness-dependent drying limitation, as the improved RF penetration promotes faster moisture migration throughout the thicker sample matrix. While the literature reports varying results depending on material properties and configuration [128], Hou et al.’s study aligns with findings in RF-dried kiwifruit [129], confirming that optimal thickness-electrode gap synergy can indeed produce this counterintuitive phenomenon.

Interestingly, drying not only removes water but also reduces pesticide residues. It has been reported that HAD can reduce 90% residues of dimethoate, diazinon, chlorpyrifos, and methidathion in dried grapes due to pesticides being degraded rapidly during the drying process as the temperature increases [130]. Zhao and colleagues also found [131] that residues of various pesticides, including dichlorvos, malathion, chlorpyrifos, triadimefon, hexaconazole, myclobutanil, kresoxim-methyl, tebuconazole, epoxiconazole, bifenthrin, and cyhalothrin were decreased by 11% to 95% in jujubes when different drying methods were used, such as FD, HAD, sun drying, and MWD. For orange slices, Acoglu and Ömeroğlu (2021) investigated the effect of four drying methods (HAD, microwave-assisted HAD, VD, and VMD) on oranges treated with four common pesticides (abamectin, buprofezin, imazalil, and thiophanate-methyl) [132]. The results showed that higher temperatures and microwave pretreatment significantly degraded pesticide residues, with hot air convective drying at 60 to 80 °C and microwave pretreatment HAD/MWHD reducing pesticide residues more than VD. For example, hot air convective drying at 80 °C reduced abamectin from 0.856 to 0.778 mg/kg, buprofezin from 2.136 to 1.976 mg/kg, imazalil from 9.803 to 9.013 mg/kg, and thiophanate-methyl from 5.286 to 4.683 mg/kg. MWHD further shortened the drying time by 43% and enhanced degradation, with thiophanate-methyl degrading faster due to its lower hydrophobicity. In contrast, VD at the same temperature resulted in higher pesticide residues, leaving up to 3.4 times higher residues than fresh samples, probably due to water loss-driven concentration, while the actual degradation is lower than in HAD due to milder thermal conditions and lack of oxidative pathways. Based on this, if pesticide reduction is the goal, combining heat and microwaves would work best and the application of drying technologies can also improve the food safety.

### 5.2. Orange Peels

Dried orange peel is the dehydrated outer skin of oranges, characterized by an intense and slightly pungent essential oil aroma, strongly zesty flavor with pronounced bitterness and mild astringency, and a hard and brittle texture featuring distinct ridges and pores, easily fragmented with rough edges and no sticky residue. In recent years, the value enhancement of orange peel (OP) has attracted much attention due to their high content of phytochemicals, which can be used as nutraceuticals and functional food. However, due to the high-water content, OP is easily spoiled by microorganisms. Therefore, drying methods are used to remove water and prevent microbial growth during industrial storage. Dried OP is not only processed into orange peel powder to be added to food products to enhance nutritional levels, but also used in functional foods for treating nausea and other ailments [133,134]. Table 2 summarizes the effects of different drying methods on the quality attributes of orange peels.

For drying orange peels, different drying methods can greatly affect the drying time. Normally, thermal drying methods take less time than non-thermal drying methods to reach a moisture content of around 10%, ranging from 7 min to over 300 min [14,15,18,29]. In contrast, FD takes a much longer time of over 1 day to reach the desired moisture content [16,136]. The main reason for this difference is the higher temperature of thermal drying. Hazra et al. (2024) found that MWD at 900 W significantly shortened the processing time to less than 5 min, achieving 0.028 g moisture/dm [14]. Similar results were obtained in a study by Wang et al. (2023), whereby MWD required a shorter period of time compared to other drying methods [15]. Meanwhile, shorter processing time means better retention of heat-sensitive bioactive and volatile compounds. As a result, it is suitable for industrial applications.

Color is another important factor to be considered as it directly influences the sensory characteristics and reflects qualitative and quantitative changes in the products. Wang et al.’s study showed that FD best preserved the color attributes, including lightness (L*) and yellowness (b*) of OP while minimizing browning, compared to other drying methods (heat pump drying, MWD, and far-infrared drying), which indicates its superior ability to maintain near-natural physicochemical properties and achieve high retention of pigments [15]. A similar result was obtained by Farahmandfar et al. (2020), freeze-dried OP had the highest lightness (L* = 48.54), yellowness (b* = 49.00) and lowest ΔE value ($∆E=\sqrt{{\left(L_{0}^{*}-L^{*}\right)}^{2}+{\left(a_{0}^{*}-a^{*}\right)}^{2}+{\left(b_{0}^{*}-b^{*}\right)}^{2}}$) [137]. The low temperature of FD made it appropriate for preserving pigments like carotenoids and flavonoids. Contrary to FD, thermal drying methods (e.g., MWD) caused more significant change to the color. According to Hazra et al. (2024), MWD 600 W resulted in the lowest L* and b* compared to fresh samples, and significantly destroyed the color (∆E=10.63), which could be caused by Maillard browning [14]. However, Phuon et al. (2020) came up with opposite results, they found that MWD led to the lowest  ∆E, which meant it caused minimal differences compared to fresh orange peel [135]. Therefore, the suitability of the drying method depends on the fresh peel and the demand.

Moreover, phytochemical retention is a key factor in determining the drying method. FD preserved bound polyphenols, while high-temperature drying resulted in their significant degradation. Phuon et al. (2020) and Farahmandfar et al. (2019) found that the TPC in freeze-dried orange peels increased by 19.5% and 252%, respectively, compared to fresh orange peels [16,135]. The reason for the higher TPC of FD samples is that they were dried at a very low temperature to retain the heat-sensitive components. Given to its ability to retain TPC, in most cases, FD can result in a higher antioxidant ability, or even increase this ability [135]. However, this does not necessarily mean that higher temperatures result in lower TPC. Comparable or better phenol retention can be obtained using other drying methods under controlled conditions. La et al. [122] reported that the highest TPC was obtained by oven drying OP at 50 °C and 70 °C, while FD had the highest TFC. Interestingly, TFC of oven drying at 70 °C was slightly lower than that of FD with no significant difference, indicating that moderate heat temperature will not significantly destroy flavonoids while inactivating enzymes (e.g., polyphenol oxidase) related to flavonoids degradation. They also found that TPC increased with temperature, suggesting that high-temperature drying can release bound phenolic acids into free form. Similar results were shown in Hazra et al. (2024)’s study [14]. They demonstrated that OP dried at 600 W of microwave power maximized the retention of bioactive compounds, with TPC of 24.65 mg GAE/g and total flavonoid content of 11.73 mg QE/g. Compared to fresh peel, MWD caused a 52–67% decrease in bound polyphenols and a 40–92% increase in free polyphenols, and a 16–33% decrease in TPC [14]. Elevated drying temperatures can release bound phenolic compounds into their free forms, leading to an increased small fraction of free phenolic compounds. These results highlight the importance of selecting appropriate drying methods to achieve the highest phenolic retention.

In addition to polyphenols, Vitamin C is also an important component of OP with antioxidant activity. Elik et al. (2023), Bozkir et al. (2020), and Deng et al. (2019) achieved 93% (the 2,6-dichloroindophenol titration method), 93.37% (measuring absorbance at 450 nm using a UV–VIS spectrophotometer), and 62% (titration with an iodate potassium solution) retention of vitamin C in dried OP using radio frequency-assisted HAD, VMD and hot air impingement drying, respectively [18,28,40]. Interestingly, dried OP also improves the extraction rate of essential oil. Farahmandfar et al. (2019) found that nine drying methods (sun, shade, oven 45 °C, oven 60 °C, vacuum oven 45 °C, vacuum oven 45 °C, microwave 360 W, microwave 600 W, and FD) had a higher yield of essential oil than fresh samples [16]. Among the drying methods, FD has the highest yield (6.90% *v*/*w*), which was almost 7-fold that of fresh peels (1.00% *v*/*w*).

Notably, pretreatments including ozone and pulsed electric field (PEF) were employed to further reduce energy consumption and retain phytochemicals in orange peel drying, which has a significant impact on product quality. Bechlin et al. (2020) found that treating OP with ozone (4 and 40 µg/L for 2 h) enhanced moisture diffusion and thus reduced drying time [27]. Color analysis revealed that ozone exposure decreased lightness (L* value decreased from 78.87 to 62.98–74.86) while increasing redness (a* value rose from 1.11 to 5.67–9.04), which may be due to oxidative reactions and microstructural changes in the peel. In terms of bioactive compounds, ozone pretreatment increased essential oil yield, reaching 4.48% with 40 µg/L ozone. Additionally, ozone treatment reduced fungal contamination by 26–29%. Another pretreatment used is PEF pretreatment with ultrasound-assisted (US) drying. Mello et al. (2021) found that although using PEF pretreatment alone did not affect processing time, combining PEF pretreatment (200 µs) with US-assisted drying (airborne ultrasound at 21.9 kHz, power density 20.5 kW/m^3^) significantly reduced the time by 33% [136]. The parameters of PEF were as follows: electrical field strength 1.20 kV/cm, pulse width 25 µs, 8 pulses, total treatment time 200 µs, specific energy 0.37 kJ/kg, tap water at 20 ± 1 °C, and conductivity 1.11 mS/cm. Subsequently, they demonstrated that low intensity PEF-US (PEF: 1.25 kV/cm, 10 Hz and a pulse width of 25 µs, and 8 pulses; US: 21.8 ± 0.4 kHz, 50 W) could shorten the time from 300 min to 180 min [22]. Importantly, the quality of dried peel was improved; 200 µs of PEF-US retained 48% of phenolic compounds (vs. 27% in conventional HAD) and minimized antioxidant degradation. However, high-intensity PEF (>1 kJ/kg) can impair drying kinetics and compound retention due to excessive cell rupture.

### 5.3. Other Products

The drying process plays a pivotal role in preserving and enhancing the quality, stability, and nutritional value of other orange-derived products, such as citrus tea, essential oils, juice powders, purees, snacks, and pomace. Most of these products are by-products from the production of oranges or orange juice. By processing and utilizing them with different drying technologies, high value utilization of oranges can be achieved, promoting the integrated use of oranges and contributing to sustainable agricultural practices.

#### 5.3.1. Citrus Tea

Citrus tea, especially orange black tea, is a popular beverage in China made from tea leaves mixed with citrus peel. This tea offers a unique synergy between the fragrant, fruity essence of OP and the flavor of black tea. In recent years, the drying method has been recognized as a critical factor influencing the sensory and nutritional quality of citrus tea. However, the conventional sunlight drying (SLD) method, although cost-effective, is highly dependent on weather conditions, leading to inconsistent quality, potential microbial contamination, or undesirable flavors like sour and astringent. Therefore, applying alternative drying methods like HAD is crucial for improving the product stability and quality of citrus tea. For example, Ni’s team (2023) reported that HAD significantly improves sensory quality, producing a stronger fruit-tea aroma and sweeter taste compared to traditional SLD. Also, HAD has a shorter drying time (20 h) than SLD (90 h) while preserving higher levels of bioactive compounds such as polyphenols, flavonoids, and hesperidin. In addition, HAD-processed tea exhibits greater antioxidant activity against FRAP, DPPH, and ABTS, as well as inhibition of α-glucosidase and α-amylase by 38.1% and 36.3% [70]. Subsequently, Ni’s team (2024) further investigated the volatile compounds of citrus tea and results showed that HAD enhances fruity and floral aromas that are produced by limonene and linalool, whereas SLD retains more woody or medicinal aromas due to prolonged oxidation. Meanwhile, temperature optimization studies indicated that HAD at 40–50 °C can contribute to lower bitterness and astringency flavor, richer free amino acids, and better comprehensive quality [121].

#### 5.3.2. Orange Juice Powder

Orange juice powder is another common orange-type product in the market and the effect of different drying methods on the physicochemical and bioactive properties of orange juice powder has been extensively studied. For instance, Castañón Rodríguez et al. (2020) investigated spray drying and showed that it was the most effective method to produce orange juice powder with low moisture (2.4%) and high polyphenol retention (573.8 mg/L) when using maltodextrin (7% *w*/*w*) and sodium alginate (0.1% *w*/*w*) as carriers at an inlet air temperature of 160 °C and an outlet temperature of 80–90 °C [123]. The spray dried powder was also had good solubility and color stability although higher temperatures slightly affected the color intensity. Foam mat drying, as explored by Nemati et al. (2022), demonstrated that higher drying temperatures (up to 80 °C for oven and 900 W for microwave) reduced moisture content and improved solubility, the powder samples with 3 mm thickness showed the highest solubility [124].

#### 5.3.3. Orange Puree

With the increasing demand for high quality, shelf stable fruit products, the effect of drying techniques on maintaining the nutritional and sensory properties of orange puree has become a critical research area. The literature review shows that FD and microwave-assisted foam mat drying are promising for achieving these goals. Specifically, Silva-Espinoza et al. [12] found that the optimal FD conditions are low pressure (5 Pa) and high shelf temperature (50 °C) for preserving bioactive compounds (e.g., vitamin C, total phenols, β-carotene) of crunchy orange puree, while also maintaining desirable structural properties such as porosity and mechanical rigidity. Meanwhile, the shorter processing time at higher temperatures (6 h at 50 °C compared to 25 h at 30 °C) minimized oxidative degradation, resulting in better nutrient retention. Additionally, the study highlighted the importance of low pressure in preventing color degradation, as higher pressures (100 Pa) led to darker and more saturated products due to increased shrinkage [138]. Similarly, Süfer et al. [139] explored the microwave-assisted foam-mat drying of bitter orange puree, evaluating the effects of microwave power levels (90, 180, and 360 W) and egg albumen (40%, 50%, and 60% *w*/*w* of fruit paste) as foam agent on drying kinetics, color, powder properties, and bioactive content. The foam was prepared by whipping egg albumen with the paste at 700 W for 10 min, achieving a foam density of 0.899–0.948 g/cm^3^ and expansion rates of 9–16%. The study demonstrated that this hybrid method combined the rapid dehydration of microwaves with the structural benefits of foam-mat drying, yielding stable foams that retain 100% stability after 3 h at 25 °C. The lowest ΔE was found in dehydrated bitter orange at 90 W with 60% egg albumen, indicating minimal browning. The resulting bitter orange powders retained significant amounts of total phenols (7.869–8.771 mg GAE/g dm) and antioxidant activity (79% retention compared to fresh fruit) [139]. However, higher microwave power levels (360 W) shorten the drying time but may also degrade thermolabile compounds like vitamin C. Based on this, FD excels in nutrient retention and structural properties, while microwave-assisted foam-mat drying provides faster processing and good foam stability.

#### 5.3.4. Orange Waste Utilization

Extraction of orange essential oil by drying the orange peel waste is one way to maximize the utilization of oranges. The research of Askin [140] showed that oven drying at 55 °C (OD55) yielded higher essential oil content (1.1%) than OD45, while MWD at 400 W (MW400) resulted in the highest yield (1.0%) among microwave methods. Notably, MW400 exhibited the highest TPC (6.99 g/kg) while unique oxygenated monoterpenes like β-linalool and neralc were found. Overall, OD55 was optimal for yield maximization, but MWD was faster (7–10 min) than OD (60–120 min) and MW400 retained more aromatic diversity [140].

Moreover, orange peel powder was another high-value utilization of orange peel. By improving the drying process, valuable compounds in orange peels can be better preserved, leading to enhanced nutritional and health benefits. Since the physicochemical and bioactive properties of orange peel powder varied with different drying techniques and parameters, Özcan et al. [141] compared MWD, ID, and OD. The results showed that ID retained the highest TPC with 177.92 mg GAE/100 g and enhanced DPPH-radical scavenging activity. OD significantly maintained the total flavonoid contents with 850.54 mg/100 g compared to 296.38 mg/100 g of fresh peels. Thus, OD60 was the most effective in improving the overall bioactive components.

In addition, the study by Afrin et al. [142] utilized orange pomace, which is a by-product of juice processing and rich in dietary fiber and bioactive compounds. Convective drying was investigated as an economical method to preserve and enhance the functionality of orange pomace for further use in food production. Researchers dried orange pomace at 50, 60, and 70 °C, respectively, and found the Modified Page model best described the drying process with effective moisture diffusivity increasing with temperature and an activation energy of 53.07 kJ/mol. Chemical composition, water holding capacity, and oil holding capacity were unaffected by temperature. Fourier transform infrared spectroscopy and X-ray diffraction analyses showed that the structure of the pomace did not change as a result of drying. The study concluded that drying at 60 °C provides an equilibrium that minimizes nutrient losses while improving functional properties, making it suitable for industrial applications.

Furthermore, Kaloudi et al. [143] demonstrated that freeze-dried OP waste effectively retained its macroporous structure, yielding a high-surface-area porous carbon material (644 m^2^/g) without chemical activation, which exhibited excellent cytocompatibility for biomedical use. And Maia et al. [144] investigated the biodrying of orange solid waste biomass under three operation modes: conventional full load (CFLT), conventional partial load (CPST), and intermittent partial load with homogenization (IPST). The study found that IPST achieved the highest moisture reduction (from 3.0 to 1.6 db) in the shortest time (48 h), outperforming CFLT (500 h) and CPST (48 h). IPST also improved heat and mass transfer mechanisms, making it a promising method for industrial-scale applications.

## 6. Conclusions

Oranges, which are widely cultivated all over the world, represent a vital agricultural commodity with immense nutritional and economic value. However, the high moisture content of fresh oranges poses significant postharvest challenges, necessitating effective preservation strategies to extend shelf life while maintaining nutritional quality. Drying technologies have emerged as a crucial solution, transforming orange slices, peels, and other by-products into stable, value-added products. VMD excels in kinetics and often in TPC/color, but AAC can be sensitive to power/pressure settings. Furthermore, innovative enhancements—such as pulsed vacuum and gas-assisted techniques—can further optimize the drying performance and product quality. Meanwhile, HAD has been revitalized through integration with auxiliary heating methods, demonstrating enhanced drying efficiency, higher nutrient retention, and better color preservation. In addition, drying processes can also reduce pesticide residues in orange slices, with heat-based and microwave-assisted methods demonstrating superior degradation efficiency compared to low-temperature techniques, thereby enhancing food safety.

For OP, thermal drying like MWD significantly accelerates the drying process, but high temperatures can degrade heat-sensitive compounds and induce browning. In contrast, FD offers better preservation of color, phytochemicals and antioxidant activity, but is time- and energy-intensive. Pretreatments like ozone and PEF enhance drying efficiency and bioactive preservation. For other orange by-products, HAD is particularly effective for products such as citrus tea and orange-based snacks, optimizing aroma and nutrient retention. FD remains the preferred method for preserving nutrients in citrus purees. Other applications benefit from specialized drying methods: MWD enables efficient essential oil extraction, spray drying ensures optimal solubility for juice powders, hybrid drying preserves quality in snack processing, and convective drying facilitates industrial-scale pomace utilization. Each application achieves optimal results through its respective tailored drying approach.

Future studies should focus on improving the efficiency of drying methods while retaining higher amounts of phytochemicals. Effective pretreatments, like controlled-atmosphere assistance, ozone, and pulsed electric field, can be introduced to valorize orange products at industrial levels, ensuring energy efficiency and cost-effectiveness. Additionally, more sensory evaluations such as the crispness of orange slices, the bitterness of orange peel and the aroma retention of citrus tea are needed to evaluate the consumer acceptability of dried orange products with altered color, texture and taste. These improvements could expand the variety of orange-derived products and enhance their nutritional profile and commercial viability, creating new opportunities for the global health food and pharmaceutical markets.

## Figures and Tables

**Table 1:** The application characteristics of different drying techniques in dried orange products.

Drying Technology	Related Product	Key Parameters	Advantages	Disadvantages
Hot Air Drying (HAD)	orange slice	Temperature: 60–80 °C Air velocity: 1–2 m/s [2,68,69]	− Low investment and operating costs − Simple and widely applicable	− Negatively affects sensory attributes (color, texture, aroma) − May degrade nutritional properties at 60–80 °C
	orange peel	Temperature: 50–60 °C Air velocity: 1–3 m/s [18,26,29]		
	citrus tea	Temperature: 40–50 °C [70,121]		
Freeze Drying (FD)	orange peel	Temperature: Pre-frozen: −80–−65 °C Dry: −60–−50 °C [14,15,16,122]	− Preserves color, aroma, flavor, and nutrients − Ideal for heat-sensitive products − Easy rehydration	− Time-consuming − High equipment and operational costs
Vacuum Drying (VD)	orange slice	Temperature: 60–80 °C Pressure: 10–30 kPa [69,76]	− Low-temperature drying prevents oxidation/browning − Retains active ingredients	− Long exposure to low pressure may cause deformation − Time-consuming
Pulsed Vacuum Drying (PVD)	orange slice	Temperature: 65–75 °C Pressure: 10–20 kPa Pulse cycle: 5:10–5:20 min [76]	− Reduces structural stress and deformation − Enhances moisture removal efficiency − Better retention of heat-sensitive plant compounds and antioxidant capacity than VD	− Excessive pressure fluctuations may cause puffing/collapse − Requires precise parameter optimization
Spray Drying	orange juice powder	Inlet temperature: 140–200 °C Outlet temperature: 40–90 °C [85,123]	− Extends shelf life of liquid foods − Produces fine, uniform powders − Fast drying (seconds to minutes)	− Not suitable for retaining heat-sensitive substances − Requires carrier agents − High energy consumption
Microwave Drying (MWD)	orange slice	Temperature of vacuum treated: 50–80 °C Pressure: 15–30 kPa Power: 90–350 W [69,76]	− High drying efficiency	− Uneven heating may cause hotspots − Can degrade color and texture at high power
	orange peel	Power: 300–900 W [14,15,16]		
	orange juice powder	Power: 360–900 W [124]		
Ozone Pretreatment	orange peel	Concentration: 4–40 µg/L [27]	− Reduces microbial load and pesticide residues − Enhances drying efficiency	− Degrades phenolics and sensitive nutrients − May cause oxidative browning − Health risks if mishandled

**Table 2:** The summary of the effects of different drying methods on the quality attributes of orange peels.

Orange Peel Samples	Drying Methods	Moisture	Color	Antioxidant Ability	Phytochemicals	Conclusion	Ref.
*Citrus sinensis* (width: 27 ± 0.577 mm, thickness: 3 ± 0.577 mm)	Freeze drying (FD): −45 °C, 14 Pa, 21 h and 5 min. Hot air oven drying: 60 °C, 300 min, air velocity of 1 m/s. Tray drying (TD): 50 °C, 350 min, air velocity of 1.5 m/s. Microwave drying (MWD): 180 W, 300 W, 600 W, 900 W.	Initial moisture content: 3.08 g/g dm Final moisture content: 0.028 g/g dm	FD showed the highest L* (74.01 ± 0.38) but largest ΔE (11.57), while MWD 180 W best preserved original color with the lowest ΔE (3.97).	The highest antioxidant activity (DPPH: 74.95%, FRAP: 292.98 µmol AAE/g) was recorded for OP dried with MWD 600 W.	FD retained the highest β-carotene 0.057 mg/g. The highest TPC (24.65 mg GAE/g) and TFC (11.73 mg QE/g) contents were in OP dried with MWD 600 W.	FD preserved β-carotene best, while MWD 600 W resulted in the highest phenolic, flavonoid, and antioxidant levels. MWD 900 W takes the shortest time (<5 min).	[14]
Brocade oranges (*Citrus sinensis*)	FD: 48 h below −60 °C, vacuum degree < 10 mbar. Heat pump drying (HPD): 16 h at (43 ± 2) °C under 90% RH. MWD: at 350 W for 35 min at 1 min intervals Far-infrared drying (FID): 20 h at (43 ± 2) °C.	Initial moisture content: not mentioned Final moisture content: <0.11 g/g dm	FD maintained the highest L* (61.86 ± 3.83) and b* (81.22 ± 4.07), while FID caused the most significant darkening (L* 36.49 ± 3.47) and color reduction, with all methods showing similar redness (a* 22–24).	DPPH (2.53 ± 0.15 mg TE/g dm) and ABTS (1.73 ± 0.11 mg TE/g dm) highest in FD. HPD had highest FRAP (50.21 ± 3.78 mg ferrous sulfate equivalent/g dm).	FD maintained the highest contents of ascorbic acid (0.46 mg/g dm), synephrine (15.58 mg/g dm), and limonin (2.34 mg/g dm). The highest TPC content (1158.00 ± 31.64 μg/g dm) and highest bioaccessibility of phenols (15.99 ± 0.75%) was observed in OP treated with MWD.	FD is more suitable for color protection. HPD and MWD are more suitable for industrial-scale drying.	[15]
*Citrus sinensis* L. (20.00 g sample)	Electrohydrodynamic (EHD) drying: a high-voltage needle-plate electrode setup, applying 9, 18, 27, 36, and 45 kV alternating current (AC) or direct current (DC). 25 ± 2 °C, 30 ± 5% RH, and wind speed of 0 m/s. Control check (CK): sun drying	Initial moisture content: 2.125 ± 0.001 g/g dm Final moisture content: 0.10 g/g dm	/	Polyphenol oxidase (PPO) and peroxidase (POD) activity significantly reduced. DC fields caused stronger inactivation than AC.	All phenolic compounds decreased due to enzymatic degradation (PPO and POD) and non-enzymatic oxidation by corona wind, but vanillic acid increased. Carotenoids: EHD drying preserved more carotenoids than CK. Decreased with higher AC voltages.	EHD drying effectively reduced drying time and affected the quality, volatile profiles, and phenolic content of citrus peel.	[35]
Orange peels (thickness: 10, 30, 50 mm)	Radio frequency-assisted hot air drying (RF-HAD): electrode gaps: 60, 70, 80 mm; 50 min, 45 ± 1 °C, 17 ± 0.5% RH, 1.5 m/s air velocity. Hot air drying (HAD): 190 min, the same conditions as RF-HAD but without RF heating.	Initial moisture content: 74.6 ± 0.5% wet basis Final moisture content: 0.09 ± 0.02 g/g dm	Fresh: L* (73.31 ± 0.02), a* (13.11 ± 0.02), b* (69.41 ± 0.11). RF-HAD: L* (76.73 ± 0.01), a* (10.06 ± 0.005), b* (67.49 ± 0.04), ΔE (4.95 ± 0.09). HAD: L* (78.98 ± 0.03), a* (7.17 ± 0.01), b* (63.84 ± 0.08), ΔE (9.92 ± 0.09).	FRAP (µmol/g dm): Fresh (153.68 ± 2.71), RF-HAD (125.87 ± 4.41), HAD (119.49 ± 2.04). RF-HAD preserved antioxidants better than HAD due to shorter drying time.	Total Phenolic Content (TPC, mg GAE/g dm) Fresh OP: 23.48 ± 0.25. RF-HAD: 22.52 ± 0.72 (no significant). HAD: 20.18 ± 0.81 (significant). Total Carotenoid Content (TCC, µg β-carotene/g dm) Fresh OP: 599.50 ± 3.28. F-HAD: 345.14 ± 4.22 (42% loss). HAD: 208.19 ± 5.72 (65% loss). Ascorbic Acid (AA, mg/100 g dm) Fresh OP: 536.07 ± 16.03. RF-HAD: 499.30 ± 10.33. HAD: 489.59 ± 2.61.	RF-HAD was superior to HAD, offering faster drying, better retention of phytochemicals, and improved color/powder properties. Optimal conditions: 70 mm electrode gap, 30 mm thickness.	[18]
Navelina (diameter: 100 μm)	Convective air-drying (CAD): 40 °C, 1800 min (no airflow), 900 min (1.6 m/s airflow); 60 °C, 720 min (no airflow), 315 min (1.6 m/s airflow); 80 °C, 360 min (no airflow), 180 min (1.6 m/s airflow).	Initial moisture content: 70 ± 1.5% Final moisture content: <10%	/	DPPH: 7.97–10.99 mg TE/g dm ABTS: 8.27–14.13 mg TE/g dm FRAP: 7.70–16.69 mg TE/g dm Highest activity: 60 °C, 1.6 m/s airflow	TPC was the highest at 60 °C, 1.6 m/s. CAD at 60 °C and 1.6 m/s retained the highest hesperidin, naringenin and naringin hydrate. Phenolic-compound content was higher when increasing the air flow. Total vitamin C content ranged from 828.36 to 1463.17 µg/g dm.	Best drying condition was 60 °C, 1.6 m/s airflow, which had the lowest degradation of phenolics, high antioxidant activity, and moderate vitamin C retention.	[29]
Newhall navel oranges (pieces of 1 cm^2^)	FD: Pre-frozen at −80 °C, dried for 48 h. Shade drying (ShD): ambient temperature (5–20 °C), 60–80% RH, 14 days. Hot-air oven drying (OD): 2 m/s airflow OD50: 50 °C, 5–10% RH, 12 h. OD70: 70 °C, 5–10% RH, 8 h. MWD: 600 W, 12 min.	Initial moisture content: not mentioned Final moisture content: 10 ± 0.5% wet basis	/	(DPPH (IC_50_, mg/mL): OD50 (1.15) > OD70 (1.18) > ShD (1.23) > MWD (1.34) > FD (1.37). ABTS Scavenging (IC_50_, mg/mL): OD50 (0.23) > ShD (0.24) > OD70 (0.28) > MWD (0.29) > FD (0.32). FRAP (µg VC/mg): OD70 (4.27) > FD (4.03) > OD50 (3.92) > MWD (3.86) > ShD (3.39). PPO activity is inactivated most with OD70.	TPC, (µM GAE/g DM): OD70 (81.36) > OD50 (78.93) > SHD (77.75) > FD (75.42) > MWD (71.72). TFC, (µM QE/g DM): FD (183.06) ≈ OD50 (182.57) > OD70 (168.14) > SHD (160.41) > MWD (139.93). Individual flavonoids (µg/mg dm): Hesperidin (highest: 36.27 in FD; lowest: 17.28 in MWD).	The results recommend the use of OD50 or OD70 for drying orange peel, both of which help the maintenance of bioactive compounds in the peel and improve its antioxidant capacity.	[122]
*Citrus sinensis* L. cv “Bollo” (pieces of around 4 cm × 0.35 cm)	Convective drying (CD): 50.14 ± 0.99 °C, air velocity 1.01 ± 0.03 m/s, 51.12 ± 1.67% RH, 4.5 h MWD: 340 W, 1 h FD: Pre-freezing: −80 °C, 20 h; −50 °C, 150–200 Pa vacuum, 5 days	Initial moisture content: 75.66–78.23% wet basis Final moisture content: MD: 11.10–11.46%, CD: 10.85–13.03%, FD: 5.49–5.79%.	MWD had the least total color difference (7.0 ± 1.5) compared to fresh samples.	Total antioxidant activity was observed to increase 44.1% compared to fresh samples, while CD increased 25.0% and MWD increased 18.1%.	FD increased TPC by 19.5% compared to fresh samples, while CD increased 6.4% and MWD decreased 13.7%.	FD present better-quality parameters.	[135]
*Citrus sinensis* var. Valencia Late (length × width × thickness: 48 ± 1 × 26 ± 1 × 3.18 ± 0.04 mm)	Pulsed electric field (PEF) Pretreatment: field strength: 1.20 kV/cm, 200 µs (0.37 kJ/kg) and 600 µs (1.12 kJ/kg). HAD: 50 ± 1 °C, 1 m/s, 3.5 ± 0.3 h. Ultrasound (US)-assisted drying: 20.5 kW/m^3^, 21.9 kHz. Combined treatments: HAD-US-200 µs, HAD-US-600 µs.	Initial moisture content: 2.70 ± 0.31 g/g dm Final moisture content: 0.6 g/g dm	/	HAD-600 µs showed a 31% retention of antioxidant capacity, which was lower than that of HAD (45%). US alone had no significant impact.	TPC retention: HAD: 27%, lowest HAD-200 µs: 48%, highest Ascorbic acid retention: similar in different conditions, 52–45%	PEF pretreatment combined with ultrasound drying better preserves color, phenolic content, and antioxidant capacity in orange peel compared to conventional drying.	[136]
Sweet orange (length × width × thickness: 10.0 × 2.5 × 0.35 cm)	Ozone concentrations: 4 µg/L and 40 µg/L for 2 h. HAD: 40 °C, 50 °C, 60 °C.	Initial moisture content: 73.89 ± 0.60% wet basis Final moisture content: 12% wet basis	L* decreased with higher ozone concentration and higher drying temperature (from 78.87 ± 0.18 to 62.98 ± 0.43). The color a* increased markedly (from 1.11 ± 0.14 to 9.04 ± 0.38), and b* decreased significantly (from 57.12 ± 0.90 to 37.90 0.89) compared to control.	DPPH radical scavenging (IC_50_, mg/mL): Best: 4.09 ± 0.12 (4 µg O_3_/L + 60 °C). Worst: 5.70 ± 0.04 (40 µg O_3_/L + 40 °C).	Phenolic Content (µg GAE/mg): Control: 9.81 ± 0.25. Treated: 6.89 ± 0.05–8.05 ± 0.14 (reduction due to ozone and drying). Essential Oil (EO) yield (g/100 g dried peel): Highest: 4.48 ± 0.07 (40 µg O_3_ L^−1^ + 40 °C). Lowest: 3.19 ± 0.01 (4 µg O_3_ L^−1^ + 60 °C). Pectin Yield (g/100 g peel): Control: 25.96 ± 0.72. Treated: 21.03 ± 0.49–24.57 ± 0.54.	Ozone pretreatment combined with HAD enhanced moisture diffusivity and EO yield but reduced phenolic content. Higher ozone (40 µg L^−1^) improved EO yield but reduced antioxidant activity. Lower ozone (4 µg L^−1^) + high drying temp (60 °C) preserved antioxidants best.	[27]
*Citrus sinensis* L. Osbeck var. Lane Late (6 ± 0.5 mm thickness)	TD: 50 °C, 300 min, air velocity 1.8 m/s, 30% RH Vacuum infrared drying (VID): 50 °C, 106 min, 500 W, 877 mbar Vacuum microwave drying (VMD): 20.67 min, 334 W, 877 mbar	Initial moisture content: 72.94% Final moisture content: TD: 9.96% VID: 7.99% VMD: 9.04%	VMD produced the brightest samples (L* = 73.64 ± 0.06) but with greatest color change (ΔE = 16.08 ± 0.08), while TD showed better color preservation (ΔE = 7.65 ± 0.10) despite lower brightness (L* = 67.92 ± 0.11), and VID significantly enhanced yellowness (b* = 62.53 ± 0.05)	/	Vitamin C (mg/100 g dm): Fresh: 292.235 ± 0.720 TD: 243.862 ± 0.671 VID: 212.050 ± 0.619 VMD: 272.862 ± 0.716 TPC (mg GAE/100 g dm): Fresh: 115.384 ± 0.320 TD: 85.677 ± 0.621 VID: 85.386 ± 0.475 VMD: 103.010 ± 0.520 Total Carotenoid Content (mg/100 g dm): Fresh: 24.775 ± 0.177 TD: 17.532 ± 0.089 VID: 15.490 ± 0.240 VMD: 20.050 ± 0.146	VMD is optimal for rapid drying and retaining antioxidants but changes color. VID is best for aroma preservation.	[28]
“Newhall” navel orange (*Citrus sinensis* Osb.) (15 mm diameter and 5 mm thickness)	Air impingement dryer: air velocity of 9 m/s 50 °C: 150 min; 55 °C: 120 min; 60 °C: 105 min; 65 °C: 90 min; 70 °C: 75 min.	Initial moisture content: 75.54 ± 1.07% wet basis Final moisture content: 10% wet basis	/	/	TPC decreased from 29.72 mg GAE/g dm to 25.73–26.72 mg GAE/g dm. TFC observed a reduction of 19.90–23.41% (no significant difference across temperatures). Ascorbic acid decreased from 86.56 mg/100 g dm to 41.01–53.87 mg/100 g.	Optimal condition: 65 °C (fast drying and best quality)	[40]
Bitter orange (*Citrus aurantium* L.)	Sun drying (SND): 15–37 °C (ambient), 48 h. Shade drying (ShD): 20 ± 5 °C (dark room), 60 h. Oven drying (OD): 45 °C,5 h; 60 °C, 4 h. Vacuum oven drying (VOD): 0.8 mbar, 45 °C, 48 h; 60 °C, 36 h. MWD: 360 W, 35 min; 600 W; 20 min. FD: pre-frozen at −18 °C, −50 °C, 24 h, 0.125 mbar.	Initial moisture content: 74.4 ± 0.6% wet basis Final moisture content: 10% wet basis	L*: FD (75.54, ± 0.08 highest), VOD 60 °C (50.24 ± 0.14, lowest). b*: FD (77.91 ± 0.06, highest), VOD 60 °C (55.75 ± 0.10, lowest) ΔE: SD (19.42 ± 0.44, lowest), VOD 60 °C (31.21 ± 0.20, highest)	DPPH radical scavenging (IC50, ppm): FD: 6916.90 (lowest IC50, highest activity). SD: 35,574.20 (weakest). FRAP (at 80,000 ppm): FD: 0.52% (highest reducing power). SND: 0.17%.	FD showed the highest EO yield (6.90% *v*/*w*). TPC (mg GAE/100 g): FD: 12.73 (highest). SND: 3.23 (lowest).	FD had the highest L*, b*, essential oil yield (6.90%), antioxidant activity, and phenolic content while exhibiting minimal color change. Sun/shade drying caused significant color degradation and low antioxidant retention.	[16]
Thomson navel orange (*Citrus sinensis* L. Osbeck)	ShD: 20 ± 5 °C, 60 h SD: direct sun/day light, 25~37 °C, 36 h Oven drying (OD): 45 °C, 5 h; 60 °C, 4 h VOD: 45 °C, up to 48 h; 60 °C, 36 h MWD: 360 W, 35 min; 600 W, 20 min FD: −50 °C, 0.125 mbar, 24 h	Initial moisture content: 77.3 ± 0.9% Final moisture content: constant weight	FD could preserve color values with the highest L* (48.54 ± 0.69), b* (49.00 ± 0.01) and lowest a* (1.79 ± 0.37). VOD at 60 °C had the lowest b* (30.28 ± 0.80) and highest ΔE (40.16 ± 1.38).	The highest antioxidant activity (lowest IC50, mg/mL) was observed in FD sample (5.00), followed by VOD 60 °C (7.24), MWD 600 W (7.50), fresh (7.86), VOD 45 °C (7.99), OD 45 °C (14.23), MWD 360 W (14.76), ShD (15.04), OD 60 °C (16.07), and SD (20.00).	TPC of drying treatments were as following order: FD > VOD 60 °C > MWD 600 W > fresh samples. FD had the highest EO yield (6.90% *v*/*w*), while the lowest EO yield was in fresh samples (1.20% *v*/*w*).	Results showed that FD performed best compared to other drying methods and could be a potential method for producing an excellent dried peel product with highest quality EO.	[137]

## Data Availability

No new data were created or analyzed in this study.

## Abbreviations

The following abbreviations are used in this manuscript:

Ascorbic Acid Content	AAC
Atmospheric Pressure Drying	APD
Browning Index	BI
Catechin Equivalent	CE
Color Difference	ΔE
Controlled Atmosphere	CAtm
Conventional Full Load	CFLT
Conventional Partial Load	CPST
Cupric Ion Reducing Antioxidant Capacity	CUPRAC
Effective Diffusivity	Deff
2,2-Diphenyl-1-Picrylhydrazyl	DPPH
Ferric Reducing Antioxidant Power	FRAP
Freeze Drying	FD
Gallic Acid Equivalent	GAE
Glass Transition Temperature	Tg
Hot Air Drying	HAD
Hot Air-Assisted Radio Frequency Drying	HA-RFD
Infrared Drying	ID
Intermittent Partial Load With Homogenization	IPST
Lightness	*L**
Microwave Drying	MWD
Microwave Pretreatment Hot Air Drying	MWHD
Microwave-Pretreated Vacuum Drying	MWVD
Moisture Content	MC
Near-Infrared	NIR
Normal Atmosphere	NAtm
Orange Peel	OP
Oven Drying	OD
Polymethoxylated Flavones	PMFs
Pulsed Electric Field	PEF
Pulsed Vacuum Drying	PVD
Radio Frequency	RF
Redness-Greenness	*a**
Rutin Equivalent	RE
Solar Drying	SD
Sunlight Drying	SLD
Total Antioxidant Capacity	TAC
Total Flavonoid Content	TFC
Total Phenolic Content	TPC
Ultrasound	US
Vacuum Drying	VD
Vacuum Infrared Drying	VID
Vacuum Microwave Drying	VMD
Yellowness-Blueness	*b**
